# Associations between Pregnancy-Related Symptoms, Serum 25(OH)D, and Physical Quality of Life in Pregnant Women

**DOI:** 10.3390/nu14030482

**Published:** 2022-01-22

**Authors:** Jennifer Woo, Susan Penckofer, Marcus Fagan, Carmen Giurgescu

**Affiliations:** 1 College of Nursing, Texas Woman’s University, Dallas, TX 76204, USA; mfagan2@twu.edu; 2 School of Nursing, Loyola University Chicago, Chicago, IL 60660, USA; spencko@luc.edu; 3 College of Nursing, University of Central Florida, Orlando, FL 32816, USA; carmen.giurgescu@ucf.edu

**Keywords:** quality of life, functional status, serum 25(OH)D, serum 25 hydroxyvitamin, vitamin D, pregnancy symptoms, Black pregnant women, fatigue, minority women

## Abstract

Vitamin D deficiency has been associated with pregnancy-related symptoms including fatigue, poor sleep quality, and musculoskeletal pain. Pregnant Black and Hispanic women are more likely to have vitamin D deficiency compared with pregnant non-Hispanic White women. Data are limited on the association of vitamin D deficiency with quality of life (QOL) among pregnant women. This study examined the association of serum 25(OH)D and pregnancy-related symptoms with QOL among pregnant predominantly minority women. Using a cross-sectional design, 119 pregnant Black and Hispanic women completed surveys and had blood drawn for serum 25(OH)D levels between 24–32 weeks gestation. Hierarchical regression analysis indicated that total pregnancy-related symptoms and serum 25(OH)D level were significant predictors of QOL, while controlling for covariates. Higher total pregnancy-related symptoms and lower serum 25(OH)D predicted poorer physical QOL. Screening for pregnancy-related symptoms and vitamin D levels among childbearing women might be important nursing interventions to improve physical QOL.

## 1. Introduction

Non-Hispanic Black and Hispanic women are at higher risk for vitamin D deficiency, defined as serum 25-hydroxyvitamin D (25(OH)D) < 20 ng/mL, compared with non-Hispanic White women [1]. Pregnancy alone is also a risk factor for vitamin D deficiency, along with obesity, darker skin pigmentation, malabsorption syndromes including bariatric surgery recipients, nephrotic syndrome, and some medications can increase risk of vitamin D deficiency [1]. Women with more melanin and darker skin tone, as the case with many women who identify as Black or African American or Hispanic, inhibit adequate vitamin D absorption because melanin blocks ultraviolet light and decreases the capacity to synthesize vitamin D [2]. Based on NHANES data with pregnant women, non-Hispanic Black and Hispanic women had lower mean serum 25(OH)D levels compared with White women (39 nmol/L (equivalent to 15.6 ng/mL); 56 nmol/L (22.4 ng/mL); and 77 nmol/L (equivalent to 30.8 ng/mL), respectively) [3]. There is evidence that people with naturally dark skin tones require at least three times longer exposure to the sun to make the same amount of vitamin D as a person with a white skin tone [1]. Physical symptoms such as fatigue, poor sleep quality, musculoskeletal pains, and urinary incontinence are more common during pregnancy due to the physiological changes of the body [4]. These symptoms have also been associated with vitamin D deficiency [5,6,7,8].

One of the most common physical ailments in pregnancy is back pain and pelvic pain as the body adjusts to the growing fetus [9]. Vitamin D deficiency has been associated with increased musculoskeletal pain, particularly back pain and decreased muscle strength in pregnancy [6,10]. In a study conducted in Sweden with women at 12 weeks’ gestation and 6–12 months postpartum, serum 25(OH)D levels were significantly lower in immigrant women (*n* = 68; mean serum 25(OH)D = 20 nmol/L ((equivalent to 8 ng/mL)) compared with a control group of native Swedish women (*n* = 51; mean 25(OH)D = 52 nmol/L (equivalent to 20 ng/mL)) [6]. Pregnant immigrant women also reported significantly higher pain scores for complaints of musculoskeletal pain compared with the control group [6]. Immigrant women in Sweden were predominantly from Somalia and had severely low serum 25(OH)D levels (<10 nmol/L or <4 ng/mL). The impact of vitamin D deficiency on musculoskeletal pain is important as it can significantly affect quality of life (QOL). In addition, there is evidence that vitamin D deficiency can also be a risk factor for the development of a rare condition in pregnancy known as pregnancy- and lactation-associated osteoporosis (PLO) that often presents with severe hip/back pain [11,12,13]. PLO “carries great physiological and psychological burdens to patients and has negative effects on quality of life and working ability” [11,13].

Poor sleep quality is another common physiologic symptom during pregnancy [14]. In a study with 2427 pregnant women, 76% of the women had poor sleep quality, 38% had insufficient sleep at night, and 49% experienced significant daytime sleepiness, suggesting that pregnant women should be screened and treated for sleep issues [14]. However, the sample included only 14.8% of women who identified as African American while 59.2% identified as Caucasian, which can limit the generalizability of the results. In a study with pregnant women (*n* = 292) and non-pregnant women healthy controls (*n* = 200), the researchers found that the risk of poor sleep increased more than two-fold in the second trimester which also correlated with poor quality of life [15]. Most recently, it was reported that vitamin D may be a significant predictor of poor sleep quality in pregnant women [8]. A study with 115 pregnant African American and Hispanic women (24–32 weeks gestation) found that lower serum 25(OH)D levels were significantly associated with worse sleep quality [8].

Similarly, poor sleep quality is inextricably linked to symptoms of fatigue which is very prevalent in women throughout pregnancy but most prominently at the beginning and end of pregnancy [16]. Fatigue is defined as a “subjective feeling of exhaustion interfering with daily activities of living” [16]. In a study conducted in Taiwan among Chinese women in their third trimester of pregnancy (*n* = 38), the researchers measured sleep objectively through actigraphy and a daily sleep diary and subjectively by self-reported measures of sleep quality [17]. Their results suggested that increased duration of sleep at night based on actigraphy measures was related with fewer symptoms of fatigue [17]. In addition, in another cohort of Chinese pregnant women (*n* = 164), poorer sleep quality based on actigraphy results and Pittsburgh Sleep Quality Index (PSQI) resulted in poorer physical and mental quality of life [18].

Pregnancy symptoms can have a significant impact on the physical well-being of childbearing women. Lagadec et al. [19] conducted a systematic review of 37 studies on factors influencing QOL in pregnant women. They found that the physical component of quality of life decreased throughout pregnancy. Symptoms such as back pain, sleep difficulties and fatigue, and nausea were noted to be factors that contributed to decreased QOL. Given that vitamin D deficiency has been associated with poor sleep quality, fatigue, and back pain which can all negatively impact health-related QOL, this is worthy of exploration. In addition, vitamin D deficiency has also been associated with poor pregnancy outcomes such as increased risk of preterm birth, preeclampsia, and gestational diabetes mellitus, which is another reason why it is important to assess any possible associations [20,21].

In addition to pregnancy-related symptoms, other factors that negatively influence physical QOL during pregnancy are gestational age, maternal age, socioeconomic status, higher body mass index (BMI), and social support [18,19,22,23]. For example, lower levels of social support have been reported to be associated with lower levels of QOL among pregnant women (*n* = 473) from Australia [22]. Specifically, in one of the few studies including a sample of non-Hispanic Black and Hispanic pregnant women (*n* = 155) who identified as Black/Jamaican (41%) and Hispanic (59%) (from Puerto Rico, Mexico, South/Latin America), lower levels of social support scores were correlated with poorer physical QOL [23]. Therefore, these factors will be included and controlled for in the present study.

Research is lacking on the association of serum 25(OH)D levels with physical QOL in pregnant women, particularly in women who identify as non-Hispanic Black and Hispanic. Much of the more current literature about quality of life in pregnant women has not included large samples of non-Hispanic Black and Hispanic women living in the United States as they have been conducted primarily in China, Turkey, Iran, Europe, or Australia [19]. Therefore, the association of serum 25(OH)D levels with pregnancy symptoms and QOL among predominantly non-Hispanic Black and Hispanic women needs to be examined given that these women are more likely to be vitamin D deficient. The purpose of this study is to examine the associations among pregnancy-related symptoms, serum 25(OH)D status, and physical QOL in a sample of predominantly non-Hispanic Black and Hispanic pregnant women.

## 2. Materials and Methods

### 2.1. Design and Sample

A sample of 125 pregnant women between 24–32 weeks gestation were recruited from a federally qualified health center (FQHC) in an urban, predominantly Medicaid patient population located in a northern latitude. However, 6 women were excluded from final analysis because pregnancy BMI was missing. Women were included in the study if they were: 18 years of age or older; spoke and read English or Spanish; had confirmed pregnancy at 24 to 32 weeks gestation; and were willing to have an extra blood draw for serum 25(OH)D level. Women were excluded from the study if they had significant mental health issues (e.g., treatment for substance abuse, psychosis, bipolar disorder, or schizophrenic episode); pre-gestational diabetes; HIV infection; or any autoimmune disorder such as multiple sclerosis or systemic lupus erythematosus.

### 2.2. Measures

#### 2.2.1. Maternal Characteristics

The first author developed a questionnaire to collect maternal sociodemographic characteristics (e.g., age, race/ethnicity, employment status, education status, household income) and obstetrical history (e.g., whether the pregnancy was planned or not).

#### 2.2.2. Serum 25(OH)D

Serum 25(OH)D levels were measured using the radioimmunoassay (RIA; Quest Diagnostics). The minimum detection limit was <5 ng/mL. Both intra-assay coefficient of variation (CV) and inter-assay CV were 5.1% [24].

#### 2.2.3. Pregnancy Symptoms

The Pregnancy Symptom Inventory (PSI) is a 41-item instrument (e.g., tiredness or fatigue, back pain, hip/pelvic pain, poor sleep on a 4-point scale (0 = never experiencing the symptom to 3 = often experiencing the symptom) to assess common symptoms that pregnant women report and its corresponding limitations on activities of daily living [25]. The tool has an option to write in a 42nd symptom if it was not listed. The instrument was created to be used as a clinical tool for healthcare providers to refer pregnant women for appropriate consultations, if needed, to identify the frequency and impact of certain pregnancy symptoms. If the participant marked “1” or greater for a pregnancy symptom, the participant then answered how limiting this pregnancy symptom was on their activities of daily living (1 = not limited at all, 2 = limited a little, and 3 = limited a lot). A total symptom frequency score was calculated using the frequency score for all 41 items, with a possible range of 0 to 123 as the maximum score. In this study, the Cronbach’s alpha for the PSI instrument was 0.90.

#### 2.2.4. Quality of Life

QOL was measured using the Standard Form-12 (SF-12) [26]. The SF-12 is an abbreviated version of the parent instrument of the Medical Outcomes Survey known as the SF-36 [27]. The SF-12 is a 12-item scale with items scored on Likert scale responses (e.g., of one of the items on SF-12, “Does your health now limit you in climbing several flights?”, “During the past 4 weeks, how much did pain interfere with your normal work (including work outside the home and housework)?) measuring two different components with six items each: physical health and mental health. The SF-12 has eight subscales: role physical, role emotional, physical function, social function, mental health, vitality, pain, and general health. The scores are standardized using norm-based references. A score of 50 (out of 100) equates an average quality of life and function whereas scores less than 30 represent functioning at a level lower than 98% of the population [26]. Higher scores represent higher levels of quality of life [26]. The SF-12 has been validated and used in pregnant non-Hispanic Black and Hispanic women including the Spanish version of SF-12 [23,28]. In the current study, the Cronbach’s α was 0.89 for the physical component score and 0.84 for the mental component score.

#### 2.2.5. Social Support

Multidimensional Scale of Perceived Social Support (MSPSS) is a 12-item instrument (e.g., “My family really tries to help me”; “I can count on my friends when things go wrong”) on a 7-point scale (1= very strongly disagree to 7 = very strongly agree) that measures social support [29]. The total score is calculated by the sum of all 12 items divided by 12; therefore, scores ranging between 1 to 2.9 are considered low levels of support, 3 to 5 are moderate levels of social support, and 5.1 to 7 is considered to be high levels of social support [29]. The MSPSS has three subscales of significant other support, family support, and friend support. The instrument has been used in low income and Spanish-speaking Hispanic samples [30]. The MSPSS was reliable among African American and Hispanic Spanish-speaking pregnant women (Cronbach’s alpha of 0.92 for the total score, 0.90 for the family subscale, 0.94 for the friend subscale, and 0.90 for the significant other subscale) [30,31]. Measurement equivalence has been demonstrated between the English version of the MSPSS and the Spanish version [31]. The overall social support tool was reliable in the current study with a Cronbach’s α of 0.96.

### 2.3. Procedures

The Institutional Review Board (IRB) at the university and clinical site in the Chicago, IL USA approved the study. Participants were recruited in multiple ways through approaching them in the waiting room at the clinical site by the investigator, flyers distributed throughout the five locations where prenatal care was being offered, and through group prenatal care visits, known as Centering Pregnancy. Women who were interested in the study completed an informed consent process in English or Spanish based on their language preference. The instruments were available in English and Spanish, and participants selected which language they preferred to complete the questionnaires. Women completed the paper questionnaires and had blood drawn by the clinical staff by venipuncture in 5 mL sterile tube. Blood was processed via centrifugation to remove serum and placed in Quest-labelled tubes with study identification numbers and processed on a daily basis with the clinic’s laboratory specimens. We attempted to have the blood drawn with their regular prenatal labs (e.g., 1 h glucose tolerance test); however, if women did not have other laboratory work done, it was an extra blood draw to participate in the study. Women were compensated USD 20 for their participation in the study.

### 2.4. Data Management and Statistical Analyses

Questionnaire data were entered into SPSS 25 (IBM) by the first author. Lab values from Quest laboratory were accessed using an online system where participants were identified with their study identification number. Six Black women had serum 25(OH)D levels 3 standard deviations above the mean, and significantly skewed the data. These six women were removed from data analysis. The final sample used for the analysis included 119 women.

All variables were examined for normality, homogeneity of variance, the presence of outliers, and missing values. Descriptive statistics were examined to describe the sample. Next, *z*-tests were conducted on the proportion of individuals who displayed pregnancy symptoms. This was done to determine if there was a difference in the proportion of individuals who displayed pregnancy symptoms between vitamin D deficient and non-deficient women. Following this, independent samples *t*-tests were conducted to compare severity of pregnancy symptoms in vitamin D deficient and non-deficient women. Women who did not experience pregnancy symptoms were excluded from the independent samples *t*-tests analyses. Bivariate analyses were conducted to examine the associations among the variables. Hierarchical regression analyses were utilized to predict physical quality of life with covariates entered in the first step (gravida, gestational age at data collection, maternal age, annual household income, and perceived social support). Pregnancy symptoms were entered in the second step and serum 25(OH)D was entered in the third step. All analyses were conducted in IBM SPSS Statistics, version 25 with a Type I error rate set to 0.05.

## 3. Results

### 3.1. Maternal Characteristics

Sixty women (50.4%) identified as Black, 49 (41.2%) as Hispanic, and 10 (8.4%) as other (see Table 1). Most participants were married or living with partners (*n* = 78; 66%), were employed full- or part-time (*n* = 65; 55%) and had vitamin D deficiency (serum 25(OH)D levels < 20 ng/mL; *n* = 70; 63.6%). The mean age of the sample was 26.6 ± 5.6 years (range of 18–43). The mean score for social support was 5.82 ± 1.5 which indicates the sample had above average levels of social support. In addition, the pre-pregnancy BMI of the sample was 28.4 kg/m^2^ ± 7.9 which, according to Centers for Disease Control, is considered overweight (see Table 2).

#### 3.1.1. Differences in Pregnancy Symptoms by Serum 25(OH)D

To check for differences in frequency of pregnancy symptoms by vitamin D deficiency, two separate analyses were conducted: *z*-tests on the proportion of individuals who displayed pregnancy symptoms (see Table 3) and independent samples *t*-tests to compare the severity of pregnancy symptoms (see Table 4). The five most common reported pregnancy symptoms in women with vitamin D deficiency were: back pain (84.9%), fatigue (82.2%), urinary frequency (80.8%), food cravings (79.5%), and headaches (78.1%). Conversely, in women without vitamin D deficiency the five most common pregnancy symptoms were: fatigue (86.5%), urinary frequency (80.8%), headache (78.8%), food cravings (76.9%), and poor sleep/hip pain (73.1%). There were no significant differences in the proportion of individuals who reported pregnancy symptoms between participants with vitamin D deficiency (serum 25(OH)D levels <20 ng/mL) and participants without vitamin D deficiency (serum 25(OH)D levels ≥20 ng/mL). However, when examining severity of symptoms, participants with vitamin D deficiency had statistically significant higher severity in fatigue (*t*_(103)_ = 2.199, *p* = 0.030, *d* = 0.42), poor sleep (*t*_(90)_ = 2.269, *p* = 0.026, *d* = 0.48), back pain (*t*_(97)_ = 3.130, *p* = 0.016, *d* = 0.64), and hip/pelvic pain (*t*_(80)_ = 2.100, *p* = 0.016, *d* = 0.46) compared with participants without vitamin D deficiency (see Table 4).

#### 3.1.2. Relationships of Study Variables with Physical Quality of Life

Correlations between demographics, pregnancy symptoms, serum 25(OH)D, and physical QOL are reported in Table 5. Results show statistically significant relationships between physical QOL and pregnancy symptoms (*r* = −0.310, *p* < 0.01), annual household income (*r* = 0.202, *p* < 0.05), pre-pregnancy BMI (*r* = −0.190, *p* < 0.05), and serum 25(OH)D (*r* = 0.268, *p* < 0.01). Non-Hispanic Black race was related to serum 25(OH)D (*r* = −0.311, *p* < 0.01). Maternal age was significantly related to gravida (*r* = 0.512, *p* < 0.01), Non-Hispanic Black race (*r* = −0.267, *p* < 0.01), pre-pregnancy BMI (*r* = 0.333, *p* < 0.01), and annual household income (*r* = 0.329, *p* < 0.01).

Hierarchical regression was conducted for physical QOL using the variables that were significantly correlated with it (i.e., pregnancy symptoms, income, pre-pregnancy BMI, and perceived social support) and serum 25(OH)D (Table 6). First, income, perceived social support, pre-pregnancy BMI, and pregnancy symptoms were entered into the model since they were found to significantly correlate with physical QOL and produced a statistically significant model, *F* _(4, 114)_ = 5.440, *p* < 0.001, *R*^2^ = 0.160. Total pregnancy symptoms (β = −0.122, $r_{s}^{2}$ = 0.599, *p* < 0.001) and income (β = 0.207, $r_{s}^{2}$ = 0.253, *p* = 0.019) were statistically significant predictors of physical QOL. Following this, serum 25(OH)D was subsequently added to the model, F _(4, 113)_ = 6.384, *p* < 0.001, *R*^2^ = 0.220, Δ*R*^2^ = 0.060, and was a statistically significant predictor, β = 0.250, $r_{s}^{2}$ = 0.327, *p* = 0.004. Additionally, total pregnancy symptoms, β = −0.294, $r_{s}^{2}$ = 0.436, *p* = 0.001, remained significant when including serum 25(OH)D in the model; however, income became non-significant. Moreover, total pregnancy symptoms ($r_{s}^{2}$ = 0.478) and serum 25(OH)D ($r_{s}^{2}$ = 0.367) had large-squared structure coefficients demonstrating that they can explain a large portion of variance in physical QOL. Interestingly enough, obesity (BMI > 30) was associated with vitamin D deficiency and poorer physical quality of life, but it was not a significant predictor in the model, therefore serum 25(OH)D and pregnancy symptom status provided greater predictive power as evidenced by their structure coefficients.

## 4. Discussion

A higher number of pregnancy-related symptoms predicted poorer physical quality of life in a sample of predominantly Black and Hispanic pregnant women in this study. The most frequently reported pregnancy-related symptoms among our sample were back pain, urinary frequency, and tiredness. The frequency of these symptoms did not differ between women who had vitamin D deficiency and women with vitamin D sufficiency. However, the pregnancy-related symptoms that most significantly impacted activities of living were tiredness, poor sleep, back pain, and hip/pelvic pain. Women who had vitamin D deficiency had significantly higher severity of these symptoms compared with women without vitamin D deficiency. These physical symptoms are common in both pregnancy and vitamin D deficiency, and significantly affected the physical quality of life among women in our study.

Other studies support the findings from this study. A study conducted in Canada of pregnant women (*n* = 245) in their third trimester (28–40 weeks) found that sleep problems were a strong predictor of poorer physical quality of life [32]. The sample was largely White (75.9%), with few minorities (6.2% identifying as Black and 2.5% as South/Latin American) represented [32]. A strength of the current study is the high representation of Black and Hispanic women living in the US, which is important since Black and Hispanic women are at greater risk for vitamin D deficiency [3,33]. The findings of a decreased physical component of quality of life in the current study are congruent with Chang et al. (2014) who studied 358 pregnant Taiwanese women over the duration of their pregnancy but saw particular decreases in physical quality of life in mid to late trimester, similar to this study [34].

Social support has been reported as an important predictor of health-related quality of life among Black pregnant women [23,35]. These results have also been reported in other samples from China, Iran, and Turkey [19]. In the current study, social support did not significantly correlate with physical quality of life. A possible explanation for this finding could be that this sample reported overall high levels of social support compared with other samples [23,35].

Vitamin D deficiency was related to physical quality of life among women from our sample. Women who had vitamin D deficiency had lower levels of physical quality of life compared women who had vitamin D sufficiency (43.42 ± 7.2 and 47.7 ± 7.2, respectively; *t_(_*_123)_ = −3.298, *p* = 0.001). Thus, screening at-risk pregnant women for vitamin D deficiency and providing vitamin D supplementation may be a feasible intervention to improve pregnancy symptoms such as back pain and poor sleep. Englund et al. conducted a vitamin D_3_ supplementation study with 25 pregnant non-Western immigrant women with severely deficient levels of serum 25(OH)D (<25 nmol/L (equivalent of 10 ng/mL)) from Sweden [36]. Women were supplemented with 1600 IUs of cholecalciferol and 1000 mg of calcium supplements daily for three months. Serum 25(OH)D was measured at baseline and after three months treatment with participants who reported improved pain scores (visual analog scale) and muscle strength once serum 25(OH)D normalized [36]. A follow-up qualitative study conducted on a subsample of these same women in the clinical trial (*n* = 6) reported these themes following supplementation: living a restrained life prior to treatment, recaptured vitality after treatment, and the need for continuity in medication [37]. This study provides some evidence that vitamin D supplementation may be a potential treatment for pregnant women, particularly Black and Hispanic women, who have deficient levels of serum 25(OH) D to address potential ailments such as back pain and poor sleep quality. Vitamin D status may an important biomarker to further investigate since it has significant implications on symptoms of muscoskeletal pain and fatigue. Clinicians should consider screening for vitamin D deficiency in high-risk pregnant women and treating with vitamin D supplementation since it could translate into improved physical quality of life, especially in non-Hispanic Black and Hispanic women.

There are limitations to this study. The sample is relatively small and homogenous with women of low socioeconomic status. Future research should include a larger sample of non-Hispanic Black and Hispanic pregnant women. Data were collected at 24–32 weeks gestation. Causative linkages between vitamin D status and poorer physical quality of life cannot be established due to the cross-sectional nature of this study. Future research should collect data at multiple time points during pregnancy and assess whatever changes in vitamin D levels across pregnancy relate to pregnancy-related symptoms and physical quality of life among pregnant women.

## 5. Conclusions

In summary, the present study revealed that low serum 25(OH) D levels and presence of a higher number of pregnancy symptoms were important predictors of poorer physical quality of life in a sample of predominantly pregnant Black and Hispanic women. Other studies have found a predictive link between poorer pregnancy and birth outcomes such as QOL of preterm birth or small size for gestational-aged infants [38,39]. Therefore, addressing both vitamin D deficiency and quality of life could be an important intervention for improving birth outcomes, especially for Black and Hispanic pregnant women. Healthcare providers need to be aware of the deterioration in sleep and health-related quality of life among pregnant women [15]. Therefore, identifying those at risk for not only vitamin D deficiency but also those experiencing more pregnancy symptoms such as poor sleep quality and back pain could provide opportunity to develop individualized treatment interventions which may include vitamin D_3_ supplementation with treatment at safe doses to ameliorate these symptoms. Vitamin D supplementation should be an important clinical consideration in at-risk populations for vitamin D deficiency to help improve physical quality of life since vitamin D status has been an important predictor of poor sleep quality and back pain.

## Figures and Tables

**Table 1:** Categorical participant characteristics (*n* = 119).

Characteristic	*n* (%)
Ethnicity	
Hispanic	49 (41.2%)
Non-Hispanic Black	60 (50.4%)
Other	10 (8.4%)
Parity	
Primipara	49 (41%)
Multiparas	70 (59%)
Relationship status	
Married	32 (27%)
Single not living with partner	41 (34%)
Single living with partner	46 (39%)
Education	
Less than high school diploma	15 (13%)
High school diploma	50 (42%)
Associate degree	7 (6%)
Some College (no degree)	34 (29%)
Bachelor’s degree and above	13 (11%)
Employment	
Full time	35 (30%)
Part time	30 (25%)
Unemployed	49 (41%)
Other	5 (4%)
Planned pregnancy	
Yes	40 (33%)
No	79 (66%)
Annual Household Income	
<USD15,000	57 (48%)
USD 15,000–USD 24,999	29 (24%)
USD 25,000–USD 39,999	19 (16%)
>USD 40,000	14 (13%)

**Table 2:** Continuous participant characteristics (*n* = 119).

Characteristic	Mean (SD)	Range
Maternal age (years)	26.6 (5.6)	18–43
Gestational age at data collection (weeks)	27.7 (2.4)	24.00–32.50
Gravida	2.9 (1.7)	1–9
Pre-pregnancy body mass index (kg/m^2^)	28.4 (7.9)	16.8–53
Serum 25(OH)D levels	19.6 (8.6)	5–54
Total Pregnancy Symptom frequency score	36.5 (18.0)	0–78
Perceived Social Support score	5.82 (1.5)	1–12.83
Quality of Life Physical Component score	45.3 (7.6)	21.89–60.88

**Table 3:** Highest reported pregnancy symptom frequency (*n* = 119).

	Vitamin D < 20 ng/mL	Vitamin D ≥ 20 ng/mL	
Symptoms	*n*/Total Who Had Symptom (%)	*n*/Total Who Had Symptom (%)	*p*
Tiredness	60/73 (82.2)	45/52 (86.5)	0.515
Food Cravings	58/73 (79.5)	40/52 (76.9)	0.338
Poor Sleep	54/73 (74.0)	38/52 (73.1)	0.912
Urinary Frequency	59/73 (80.8)	42/52 (80.8)	0.992
Back Pain	62/73 (84.9)	37/52 (71.2)	0.061
Hip/Pelvic Pain	44/73 (60.3)	38/52 (73.1)	0.136
Breast Pain	50/73 (68.5)	35/52 (67.3)	0.888
Headache	57/73 (78.1)	41/52 (78.8)	0.920
Sore Nipples	53/73 (72.6)	33/52 (63.5)	0.275
Shortness of Breath	41/73 (56.2)	30/52 (57.7)	0.865

**Table 4:** Means of limitation scores by vitamin D groups (*n* = 119).

	25(OH)D < 20 ng/mL	25(OH)D ≥ 20 ng/mL		
Symptom	M (SD)	M (SD)	*p*	Cohen’s *d*
Tiredness	2.28 (0.7)	2.00 (0.6)	0.030	0.42
Poor Sleep	2.28 (0.7)	1.92 (0.7)	0.026	0.48
Back Pain	2.39 (0.7)	1.89 (0.8)	0.002	0.64
Hip/pelvic Pain	2.16 (0.7)	1.82 (0.7)	0.039	0.46
Headache	1.86 (0.7)	1.85 (0.7)	0.968	0.01
Shortness of Breath	1.88 (0.8)	1.6 (0.6)	0.082	0.43

**Table 5:** Correlation matrix for maternal characteristics, pregnancy symptoms, Serum 25(OH) D, and physical quality of life (*n* = 119).

	1	2	3	4	5	6	7	8	9	10
Physical Quality of Life	-									
Gravida	−0.024	-								
Gestation Age at Data Collection	−0.086	0.105	-							
Non-Hispanic Black	−0.142	0.025	0.065	-						
Pre-Pregnancy BMI	−0.190 *	0.117	0.092	−0.140	-					
Maternal Age	0.063	0.512 **	−0.056	−0.267 **	0.333 **	-				
Annual Household Income	0.202 *	−0.099	−0.012	−0.245 **	−0.052	0.329 **	-			
Perceived Social Support	−0.084	−0.111	−0.070	−0.183 *	0.238 **	0.084	0.137	-		
Pregnancy Symptoms	−0.310 **	−0.097	−0.149	0.044	0.137	0.045	0.010	0.076	-	
Serum 25(OH)D	0.268 **	−0.185 *	−0.028	−0.311 **	−0.040	0.086	0.169	0.108	0.017	-

* *p* < 0.05; ** *p* < 0.01.

**Table 6:** Hierarchical regression model of pregnancy symptoms and Serum D on physical quality of life (*n* = 119).

	$R^{2}\;$	Δ*R*^2^	*b*	β	$r_{s}$	$r_{s}^{2}$	*p*
Step 1	0.160						<0.001 **
Perceived Social Support			−0.301	−0.060	−0.209	0.043	0.503
Annual Household Income			0.914	0.207	0.504	0.253	0.019 *
Pre-Pregnancy BMI			−0.121	−0.125	−0.474	0.224	0.165
Pregnancy Symptoms			−0.122	−0.290	−0.774	0.599	0.001 **
Step 2	0.220	0.060 **					0.004 **
Perceived Social Support			−0.426	−0.085	−0.178	0.031	0.330
Annual Household Income			0.746	0.169	0.430	0.185	0.050 *
Pre Pregnancy BMI			−0.106	−0.110	−0.405	0.164	0.206
Pregnancy Symptoms			−0.124	−0.294	−0.660	0.436	0.001 **
Serum 25(OH)D			0.220	0.250	0.572	0.327	0.004 **

*b* = unstandardized coefficients; *SE* = standard error; β = standardized coefficients; $r_{s}$ = structure coefficients; $r_{s}^{2}$ = squared structure coefficients. * *p* < 0.05; ** *p* < 0.01.

## Data Availability

The data is available from the corresponding author upon signina data use agreement with Loyola University Chicago.
